# Successful retrieval of a fractured biliary guidewire using a newly developed endoscopic tapered sheath

**DOI:** 10.1055/a-2073-5147

**Published:** 2023-05-10

**Authors:** Yuki Mori, Akira Kurita

**Affiliations:** 1 Department of Gastroenterology and Hepatology, Medical Research Institute Kitano Hospital, PIIF Tazuke-Kofukai, Kitano, Osaka, Japan; 2 Department of Gastroenterology and Hepatology, Rakuwakai Otowa Hospital, Kyoto, Japan

An 81-year-old man with Bismuth–Corlette type II perihilar cholangiocarcinoma ( Fig. 1 ) underwent endoscopic retrograde cholangiopancreatography (ERCP). Cholangiography showed a hilar stricture that was so severe that the biopsy forceps could not be passed alongside the tumor ( Fig. 2 a ). During this ERCP, a guidewire was fractured. An endoscopic nasobiliary drainage tube was inserted into the right anterior duct, without removal of the fragment being attempted ( Fig. 2 b ).

**Fig. 1:**
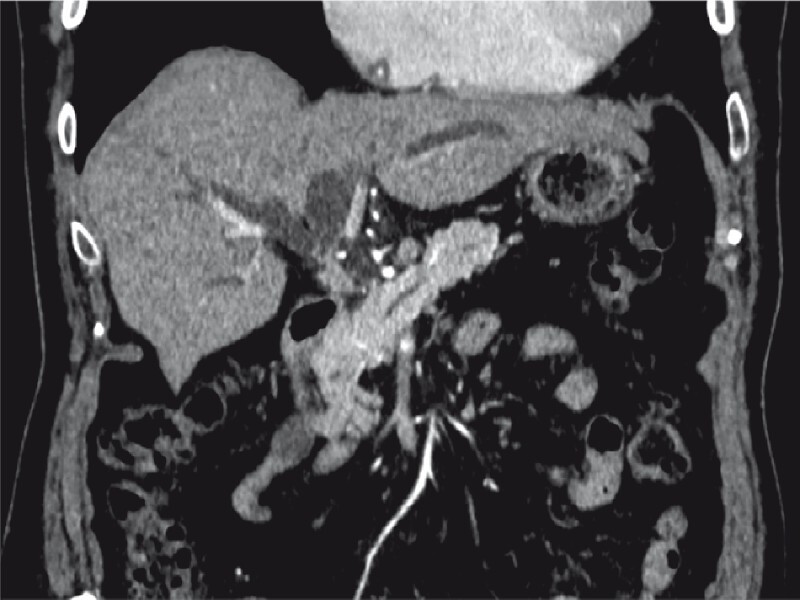
Contrast-enhanced computed tomography scan showing a Bismuth–Corlette type II perihilar cholangiocarcinoma.

**Fig. 2:**
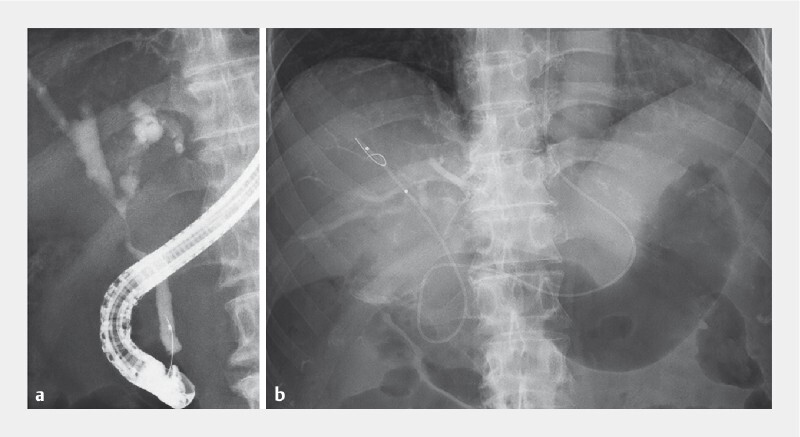
Fluoroscopic images during endoscopic retrograde cholangiopancreatography showing: **a** a severe hilar stricture; **b** the guidewire fragment located beyond the malignant biliary obstruction.

A second ERCP was subsequently performed and, after insertion of a 0.025‐inch angle‐tip guidewire (Visiglide2; Olympus Medical Systems), an ERCP catheter (MTW Endoskopie, Wesel, Germany) was placed over the guidewire fragment. An attempt to remove it using a basket catheter through the MTW catheter was however unsuccessful. As an alternative, a newly developed endoscopic tapered sheath (EndoSheather; Piolax, Inc., Kanagawa, Japan) was inserted through the hilar stricture, without dilation being required. The inner catheter and VisiGlide2 were then removed, leaving the outer sheath in position. Intraductal cholangioscopy forceps (SpyBite Max; Boston Scientific, Natick, Massachusetts, USA) were inserted through the outer sheath ( Fig. 3 ), which enabled the guidewire fragment finally to be removed ( Fig. 4 ; Video 1 ).

**Fig. 3:**
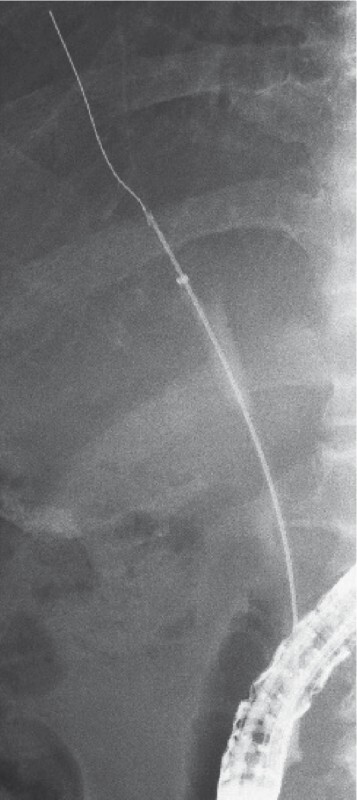
Fluoroscopic image showing the intraductal cholangioscopy forceps being inserted through the novel tapered endoscopic sheath to remove the guidewire fragment.

**Fig. 4:**
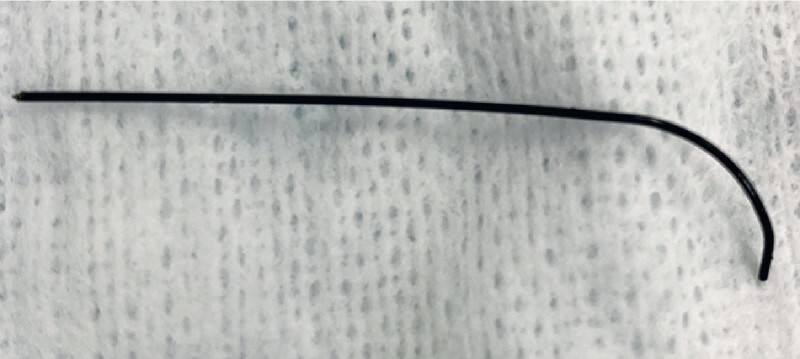
Photograph of the removed guidewire fragment.

**Video 1** A new technique is used to remove a fractured guidewire located in a peripheral bile duct beyond a malignant biliary obstruction using a newly developed endoscopic tapered sheath.


Guidewire fracture is a rare complication of ERCP. Although there are several reports of the removal of a fractured guidewire fragment, a fragment that is situated beyond a malignant biliary obstruction is considered difficult to remove 1 2 3 4 . We have described the first case in which a guidewire fragment that was beyond a malignant stricture was successfully removed using this novel tapered sheath and cholangioscopy forceps. Even with severe bile duct stenosis, as seen in this case, it appears possible to safely and easily remove a fragment from a peripheral bile duct using this method. This new technique may in future become one of the standard methods for removal of guidewire fragments.

Endoscopy_UCTN_Code_TTT_1AR_2AK

## Notes

Endoscopy E-Videos

https://eref.thieme.de/e-videos
*E-Videos* is an open access online section of the journal *Endoscopy* , reporting on interesting cases and new techniques in gastroenterological endoscopy. All papers include a high-quality video and are published with a Creative Commons CC-BY license. Endoscopy E-Videos qualify for HINARI discounts and waivers and eligibility is automatically checked during the submission process. We grant 100% waivers to articles whose corresponding authors are based in Group A countries and 50% waivers to those who are based in Group B countries as classified by Research4Life (see: https://www.research4life.org/access/eligibility/ ). This section has its own submission website at https://mc.manuscriptcentral.com/e-videos
